# Supplementary material for: Acquisition of epithelial plasticity in human chronic liver disease
Source: Nature. 2024 May 22;630(8015):166–73. doi: 10.1038/s41586-024-07465-2 (PMC11153150; doi:10.1038/s41586-024-07465-2)
Supplement: Supplementary file 1 — Reporting Summary [file 41586_2024_7465_MOESM1_ESM.pdf]

nature portfolio

Corresponding author(s): Ludovic Vallier
Last updated by author(s): 02/04/2023

Reporting Summary

Nature Portfolio wishes to improve the reproducibility of the work that we publish. This form provides structure for consistency and transparency in reporting. For further information on Nature Portfolio policies, see our Editorial Policies and the Editorial Policy Checklist.

Statistics

For all statistical analyses, confirm that the following items are present in the figure legend, table legend, main text, or Methods section.

n/a	Confirmed	
☐	☒	The exact sample size (*n*) for each experimental group/condition, given as a discrete number and unit of measurement
☐	☒	A statement on whether measurements were taken from distinct samples or whether the same sample was measured repeatedly
☐	☒	The statistical test(s) used AND whether they are one- or two-sided<br>*Only common tests should be described solely by name; describe more complex techniques in the Methods section.*
☒	☐	A description of all covariates tested
☐	☒	A description of any assumptions or corrections, such as tests of normality and adjustment for multiple comparisons
☒	☐	A full description of the statistical parameters including central tendency (e.g. means) or other basic estimates (e.g. regression coefficient) AND variation (e.g. standard deviation) or associated estimates of uncertainty (e.g. confidence intervals)
☐	☒	For null hypothesis testing, the test statistic (e.g. *F*, *t*, *r*) with confidence intervals, effect sizes, degrees of freedom and *P* value noted<br>*Give P values as exact values whenever suitable.*
☒	☐	For Bayesian analysis, information on the choice of priors and Markov chain Monte Carlo settings
☒	☐	For hierarchical and complex designs, identification of the appropriate level for tests and full reporting of outcomes
☒	☐	Estimates of effect sizes (e.g. Cohen's *d*, Pearson's *r*), indicating how they were calculated

Our web collection on statistics for biologists contains articles on many of the points above.

Software and code

Policy information about availability of computer code

Data collection	Zen 2011 SP7 on Zeiss 710, 880 or 980 confocal microscope. QuantStudio 5 384 well block (Thermo Fisher).
Data analysis	For snRNAseq, pre-processing of raw count matrices was performed using Seurat (v4.0.3) [doi:10.1016/j.cell.2021.04.048]. Gene expression values were normalised for library size using sctransform version 0.3 (doi: 10.1186/s13059-019-1874-1). The clustering parameters used were identified by evaluating the resulting cluster stability using ClustAssess doi:(10.1101/2022.01.31.478592). Data was integrated using Harmony version 0.1.1. doi: 10.1038/s41592-019-0619-0). Velocyto (v0.17.17), and velocyto.R (v0.6) were used to estimate RNA velocity based on prevalence of spliced and unspliced mRNA [doi: 10.1038/s41586-018-0414-6]. Gene set enrichment analysis (GSEA) was done using gprofiler2 (version 0.2.0). For snRNAseq Scripts for all bioinformatics analyses carried out are made available at https://github.com/Core-Bioinformatics/NAFLD-NASH.<br>Prism 9 was used for creating graphs and statistical analyses. Images were analysed using Imaris 9.7.1.

For manuscripts utilizing custom algorithms or software that are central to the research but not yet described in published literature, software must be made available to editors and reviewers. We strongly encourage code deposition in a community repository (e.g. GitHub). See the Nature Portfolio guidelines for submitting code & software for further information.

Data

Policy information about availability of data

All manuscripts must include a data availability statement. This statement should provide the following information, where applicable:

- Accession codes, unique identifiers, or web links for publicly available datasets
- A description of any restrictions on data availability
- For clinical datasets or third party data, please ensure that the statement adheres to our policy

The sequencing data and raw expression matrix is available on the Gene Expression Omnibus (GEO) series entry GSE202379.
R Shiny apps illustrating the analysis can be found at https://bioinf.stemcells.cam.ac.uk/shiny/vallier/LiverPlasticity_GribbenGalanakis2023/

Human research participants

Policy information about studies involving human research participants and Sex and Gender in Research.

Reporting on sex and gender	30 males and 17 females were included in the dataset. Sex and age of patients are included Extended Data Table 1-2.
Population characteristics	Samples from 47 patients were included in the snRNAseq data and organoids were derived from 10 patients. Population information is provided in extended tables 1-3.
Recruitment	Patients were referred to Addenbrookes Hospital liver unit for liver biopsy due to suspected non-alcoholic fatty liver disease. Screening criteria: 18 years old or above, suspected NAFLD on referral, alcohol intake of less than 14 units / week.
Ethics oversight	Biopsy collection and processing of human samples was carried out under ethics approved by Addenbrookes hospital REC 18/WM/0397. The study met all criteria for responsible use of human tissue that is used in the UK. All patients were offered the patient information sheet and provided informed consent. Healthy deceased transplant organ donor tissue and explants were taken under ethics approved by NRES Committee East of England - Cambridge South (REC number REC 15/EE/152). All patients provided informed consent.

Note that full information on the approval of the study protocol must also be provided in the manuscript.

Field-specific reporting

Please select the one below that is the best fit for your research. If you are not sure, read the appropriate sections before making your selection.

☒ Life sciences ☐ Behavioural & social sciences ☐ Ecological, evolutionary & environmental sciences

For a reference copy of the document with all sections, see nature.com/documents/nr-reporting-summary-flat.pdf

Life sciences study design

All studies must disclose on these points even when the disclosure is negative.

Sample size	No statistical analysis were performed to predetermine sample size. For snRNAseq all biopsies which were collected and successfully processed for snRNAseq based on bioinformatics QC were included in the dataset. All were included to allow for representation of all disease stages. For organoid experiments at least an n of 3 (where n is a different organoid line) were used.
Data exclusions	No data was excluded
Replication	For organoid experiments at least an n of 3 (where n is a different organoid line) were used. Details of n are given in figure legends.
Randomization	Patients were categorized by disease stage based on histology. For organoid work, cells were plated in different wells of a 24 well plate and were randomly allocated to experimental groups (control and treatments).
Blinding	Blinding was not possible for sample processing due to the logistics of patient sample collection and tissue processing.

Reporting for specific materials, systems and methods

We require information from authors about some types of materials, experimental systems and methods used in many studies. Here, indicate whether each material, system or method listed is relevant to your study. If you are not sure if a list item applies to your research, read the appropriate section before selecting a response.

Materials & experimental systems

n/a	Involved in the study
☐	☒ Antibodies
☐	☒ Eukaryotic cell lines
☒	☐ Palaeontology and archaeology
☒	☐ Animals and other organisms
☒	☐ Clinical data
☒	☐ Dual use research of concern

Methods

n/a	Involved in the study
☒	☐ ChIP-seq
☒	☐ Flow cytometry
☒	☐ MRI-based neuroimaging

Antibodies

Antibodies used

Albumin (Bethyl A80-229A)
K19 (abcam ab7754)
K7 (abcam ab68459)
MRP2 (abcam ab3373)
GLUL (abcam ab125724)
ASS1 (Cambridge Bioscience HPA020896)
SOX4 (ab86809)
K23 (Cambridge Bioscience HPA016959)
GSTA1 (abcam ab53940)
HepPar1 (Agilent Dako clone 0CH1E5)
KLF6 (Sigma HPA069585)
NCAM1 (Sigma HPA039835)
Alexa fluor 488 donkey anti-goat (Invitrogen A11055)
Alexa fluor 568 donkey anti-rabbit (Invitrogen A10042)
Alexa fluor 647 donkey anti-mouse (Invitrogen A31571).
FlexAble CoraLite® Plus 647 Antibody Labeling Kit for Rabbit IgG
FlexAble CoraLite® Plus 647 Antibody Labeling Kit for Mouse IgG1
FlexAble CoraLite® Plus 555 Antibody Labeling Kit for Rabbit IgG
FlexAble CoraLite® Plus 555 Antibody Labeling Kit for Mouse IgG1
FlexAble CoraLite® Plus 488 Antibody Labeling Kit for Mouse IgG1
FlexAble CoraLite® Plus 488 Antibody Labeling Kit for Rabbit IgG

Validation

Albumin (Bethyl A80-229A) https://www.thermofisher.com/antibody/product/Human-Albumin-Antibody-Polyclonal/A80-229A
K19 (abcam ab7754) https://www.abcam.com/cytokeratin-19-antibody-a53-ba2-cytoskeleton-marker-ab7754.html
K7 (abcam ab68459) https://www.abcam.com/cytokeratin-7-antibody-epr1619y-cytoskeleton-marker-ab68459.html
MRP2 (abcam ab3373) https://www.abcam.com/mrp2-antibody-m2-iii-6-ab3373.html
GLUL (abcam ab125724) https://www.abcam.com/glutamine-synthetase-antibody-6glutamine-synthetase-ab125724.html
ASS1 (Cambridge Bioscience HPA020896) https://www.bioscience.co.uk/product~683967. Validated as part of the Human Protein Atlas.
SOX4 (ab86809) https://www.abcam.com/sox4-antibody-ab86809.html.
K23 (Cambridge Bioscience HPA016959) https://www.bioscience.co.uk/product~682837. Validated as part of the Human Protein Atlas.
GSTA1 (abcam ab53940) https://www.abcam.com/gsta1-antibody-ab53940.html
HepPar1 (Agilent Dako clone 0CH1E5) https://www.agilent.com/en/product/immunohistochemistry/antibodies-controls/primary-antibodies/hepatocyte-%28dako-omnis%29-76237
KLF6 (Sigma HPA069585) https://www.sigmaaldrich.com/GB/en/product/sigma/hpa069585
NCAM1 (Sigma HPA039835) https://www.sigmaaldrich.com/GB/en/product/sigma/hpa039835
Alexa fluor 488 donkey anti-goat (Invitrogen A11055) https://www.thermofisher.com/antibody/product/Donkey-anti-Goat-IgG-H-L-Cross-Adsorbed-Secondary-Antibody-Polyclonal/A-11055
Alexa fluor 568 donkey anti-rabbit (Invitrogen A10042) https://www.thermofisher.com/antibody/product/Donkey-anti-Rabbit-IgG-H-L-Highly-Cross-Adsorbed-Secondary-Antibody-Polyclonal/A10042
Alexa fluor 647 donkey anti-mouse (Invitrogen A31571). https://www.thermofisher.com/antibody/product/Donkey-anti-Mouse-IgG-H-L-Highly-Cross-Adsorbed-Secondary-Antibody-Polyclonal/A-31571
https://www.ptglab.com/products/FlexAble-CoraLite-Plus-647-Antibody-Labeling-Kit-for-Rabbit-IgG-KFA003.htm#:~:text=Product%20Information-,FlexAble%20CoraLite®%20Plus%20647%20Antibody%20Labeling%20Kit%20for%20Rabbit,primary%20antibodies%20from%20any%20supplier.
https://www.ptglab.com/products/FlexAble-CoraLite-Plus-647-Antibody-Labeling-Kit-for-Mouse-IgG1-KFA023.htm#:~:text=Product%20Information-,FlexAble%20CoraLite®%20Plus%20647%20Antibody%20Labeling%20Kit%20for%20Mouse,primary%20antibodies%20from%20any%20supplier.
https://www.ptglab.com/products/FlexAble-CoraLite-Plus-555-Antibody-Labeling-Kit-for-Rabbit-IgG-KFA002.htm
https://www.fishersci.co.uk/shop/products/flexable-coralite-plus-550-antibody-labeling-kit-mouse-igg1-3/p-7227370
https://www.ptglab.com/products/FlexAble-CoraLite-488-Antibody-Labeling-Kit-for-Rabbit-IgG-KFA001.htm
https://www.ptglab.com/products/FlexAble-CoraLite-488-Antibody-Labeling-Kit-for-Rabbit-IgG-KFA001.htm

Eukaryotic cell lines

Policy information about cell lines and Sex and Gender in Research

Cell line source(s)	12 organoid lines were derived in this study (from 8 males and 4 females). Age and sex are listed in extended data table 4
Authentication	Organoid lines were derived from samples collected straight from clinic.
Mycoplasma contamination	All lines tested negative for mycoplasma contamination.
Commonly misidentified lines (See ICLAC register)	None were used in this study
