# Supplementary material for: Acquisition of epithelial plasticity in human chronic liver disease
Source: Nature. 2024 May 22;630(8015):166–73. doi: 10.1038/s41586-024-07465-2 (PMC11153150; doi:10.1038/s41586-024-07465-2)
Supplement: Supplementary file 3 — This file contains Supplementary Tables 1–4. [file 41586_2024_7465_MOESM3_ESM.pdf]

Patient ID	Disease status	SAF Score	Sex	Age	BMI	Ethnic group
3	MASH w/o cirrhosis	S2A3F1	M	56	38.01	White British
6	MASH w/o cirrhosis	S2A3F1	M	46	41.86	White British
7	MASH w/o cirrhosis	S3A4F2	M	47	34.46	White British
8	MASH w/o cirrhosis	S2A4F3	M	65	32.46	White British
9	MASH w/o cirrhosis	S2A2F1	M	50	36.68	White British
11	MASH w/o cirrhosis	S2A4F3	M	63	44.08	White British
15	MASH w/o cirrhosis	S2A4F3	M	65	44.9	White British
16	MASH w/o cirrhosis	S1A4F3	F	70	27.41	White British
19	MASH w/o cirrhosis	S1A4F3	M	76	26.62	White British
20	MASH w/o cirrhosis	S2A3F3	F	71	48.96	White British
21	MASH w/o cirrhosis	S2A3F2	M	60	34.46	White British
48	MASH w/o cirrhosis	S1A2F2	F	44	30.8	White British
49	MASH w/o cirrhosis	S1A3F2	F	55	31.35	White British
50	MASH w/o cirrhosis	S3A3F2	M	45	29.5	White British
52	MASH w/o cirrhosis	S2A2F1	M	49	31.96	White British
53	MASH w/o cirrhosis	S2A4F3	M	57	28.37	White British
54	MASH w/o cirrhosis	S3A3F2	F	61	37.73	White British
57	MASH w/o cirrhosis	S2A3F2	F	53	38.94	White British
62	MASH w/o cirrhosis	S2A3F3	M	60	34.41	White British
64	MASH w/o cirrhosis	S3A3F1	F	55	37.41	White British
67	MASH w/o cirrhosis	S2A2F3	F	67	28.83	White British
70	MASH w/o cirrhosis	S2A3F3	M	59	35.49	White British
71	MASH w/o cirrhosis	S2A4F2	F	32	42.79	White British
72	MASH w/o cirrhosis	S2A3F3	M	41	33.16	White British
73	MASH w/o cirrhosis	S2A3F1	F	59	37.08	White British
76	MASH w/o cirrhosis	S1A3F2	M	55	34.81	White British
77	MASH w/o cirrhosis	S1A3F2	F	62	31.25	White British
83	MASH w/o cirrhosis	S2A4F2	M	56	33.5	White British
12	MASH with cirrhosis	S2A3F4	F	48	45.9	White British
22	MASH with cirrhosis	S1A3F4	M	55	29.4	other white background (Portugal)
68	MASH with cirrhosis	S1A3F4	F	66	42.91	White British
75	MASH with cirrhosis	S1A3F4	M	60	37.49	White British
51	MASLD	S1A1F1	M	57	38.84	White British
55	MASLD	S1A1F2	M	21	45.14	White British
56	MASLD	S1A1F1	F	47	42.95	White British
60	MASLD	S1A1F0	M	53	33.53	White British
78	MASLD	S1A1F0	M	53	32.48	White British
84	MASLD	S1A0F3	M	56	28	White British
CL103	end stage	end stage	M	59	43.57	White British
CL104	end stage	end stage	F	59	32	White British
CL17	end stage	end stage	M	72	26.36	White British
CL18	end stage	end stage	F	68	30.13	White British
CL16	end stage	end stage	M	63	53	White British

Supplementary table 1

Demographics of patients included in snRNAseq.

Patient ID	Disease status	SAF Score	Gender	Age	BMI	Ethnic group	Notes	Liver function tests
30	Healthy control	S0A1F0	M	49	26.16	White British	Screening biopsy - No MASLD/MASH diagnosis	Bilirubin (0 – 20 umol/L) 17<br>ALP (30 – 130 U/L) 65<br>ALT(10 – 49 U/L) 46<br>Aspartate Transaminase (<=34 U/L)
98	Healthy control	S1A1F1	M	57	33.9	Nigerian	Screening biopsy -No MASLD/MASH diagnosis. Diagnosed with biliary stones	Bilirubin (0 – 20 umol/L) 16 ALP (30 – 130 U/L) 45 ALT(10 – 49 U/L) 58 Aspartate Transaminase (<=34 U/L) 34
HL1	Healthy control	healthy control	M	38	32	White British	Reason for liver transplant rejection: Congenital heart disease altered vasculature anatomy and patchy liver prefusion.	Bilirubin (0 – 20 umol/L) 5<br>ALP (30 – 130 U/L) 91<br>ALT(10 – 49 U/L) 28
HL2	Healthy control	healthy control	F	74	n/a	White British ethnicity	Reason for liver transplant rejection: Combination of alcohol intake and liver lesion on hepatic segment 6.	Bilirubin (0 – 20 umol/L) 11<br>ALP (30 – 130 U/L) 84<br>ALT(10 – 49 U/L) 11

Supplementary table 2

Demographics of healthy deceased transplant organ donors included in snRNAseq with notes on samples.

**Hepatocytes**	**Cholangiocytes**	**Stellate cells**	**Endothelial**	**Lymphocytes**	**Macrophages**	**Neutrophils**	**B cells 1**	**B cells 2**
ASGR1	CFTR	DCN	KDR	IL7R	NDST3	FCN1	BANK1	IGKC
TFR2	BICC1	COL3A1	FCN3	CD96	MARCO	ITGAX	PAX5	FCRL5
APOC1	PKHD1	PTH1R	FLT1	BCL11B	CD163		IGKC	
ADH4	CTNND2	ADAMTSL2	OIT3	THEMIS	MSR1		FCRL5	
HPX	RAYL	CCBE1	STAB2	CD247	GAS7			
C3	KRT19	ADAMTS2	TEK	PTPRC	CD5L			
FGB	KRT7	ADAMTSL1	PTPRB					
PLG	SOX9		FCN2					
FYB2	SPP1							
APOB	MUC1							
GHR	MUC6							
TENM2								
CYP3A4								
CPS1								
ASS1								
GLUL								

Supplementary table 3

Markers used in annotation of cell types in snRNAseq data.

Organoid lines	Disease stage	Age	Gender
CL2	End stage – explant	54	male
CL3	End stage – explant	67	male
CL17	End stage – explant	71	male
CL18	End stage – explant	67	female
CL103	End stage – explant	58	male
CL104	End stage – explant	59	female
CL109	End stage – explant	61	male
CL117	End stage – explant	55	female
HL1	Healthy – cadaveric donor	38	male
HL4	Healthy – cadaveric donor	41	Male
HL6	Healthy – cadaveric donor	37	Female
HL3	Healthy – cadaveric donor	44	Male

Supplementary table 4
Demographics of patients from which organoid lines were generated.
